# A Mutation in *PMP2* Causes Dominant Demyelinating Charcot-Marie-Tooth Neuropathy

**DOI:** 10.1371/journal.pgen.1005829

**Published:** 2016-02-01

**Authors:** Young Bin Hong, Jaesoon Joo, Young Se Hyun, Geon Kwak, Yu-Ri Choi, Ha Kyung Yeo, Dong Hwan Jwa, Eun Ja Kim, Won Min Mo, Soo Hyun Nam, Sung Min Kim, Jeong Hyun Yoo, Heasoo Koo, Hwan Tae Park, Ki Wha Chung, Byung-Ok Choi

**Affiliations:** 1 Stem Cell and Regenerative Medicine Center, Samsung Medical Center, Seoul, Korea; 2 Department of Health Sciences and Technology, Samsung Advanced Institute for Health Science and Technology, Sungkyunkwan University, Seoul, Korea; 3 Department of Biological Science, Kongju National University, Gongju, Korea; 4 Department of Neurology, Samsung Medical Center, Sungkyunkwan University School of Medicine, Seoul, Korea; 5 Neuroscience Center, Samsung Medical Center, Seoul, Korea; 6 Department of Radiology, Ewha Womans University, School of Medicine, Seoul, Korea; 7 Department of Pathology, Ewha Womans University, School of Medicine, Seoul, Korea; 8 Department of Physiology, College of Medicine, Dong-A University, Busan, Korea; Baylor College of Medicine, UNITED STATES

## Abstract

Charcot-Marie-Tooth disease (CMT) is a heterogeneous group of peripheral neuropathies with diverse genetic causes. In this study, we identified p.I43N mutation in *PMP2* from a family exhibiting autosomal dominant demyelinating CMT neuropathy by whole exome sequencing and characterized the clinical features. The age at onset was the first to second decades and muscle atrophy started in the distal portion of the leg. Predominant fatty replacement in the anterior and lateral compartment was similar to that in CMT1A caused by *PMP22* duplication. Sural nerve biopsy showed onion bulbs and degenerating fibers with various myelin abnormalities. The relevance of *PMP2* mutation as a genetic cause of dominant CMT1 was assessed using transgenic mouse models. Transgenic mice expressing wild type or mutant (p.I43N) *PMP2* exhibited abnormal motor function. Electrophysiological data revealed that both mice had reduced motor nerve conduction velocities (MNCV). Electron microscopy revealed that demyelinating fibers and internodal lengths were shortened in both transgenic mice. These data imply that overexpression of wild type as well as mutant *PMP2* also causes the CMT1 phenotype, which has been documented in the *PMP22*. This report might expand the genetic and clinical features of CMT and a further mechanism study will enhance our understanding of *PMP2*-associated peripheral neuropathy.

## Author Summary

Isolation of causative mutation is still challenging in genetic diseases with a variety of genetic causes. We discovered a mutation in a novel gene from a family exhibiting a peripheral neuropathy by virtue of next-generation sequencing. Although the family shows characteristic clinical features of hereditary motor and sensory neuropathy, we could not find a mutation from well-known genes. To demonstrate the clinical relevance of the novel gene, we generated transgenic mice, which carry the patients’ mutation within their chromosome. The transgenic mice exhibited the same phenotype as the patients including peripheral neuropathic symptoms and reduced locomotor function. We also observed the affected peripheral nervous system through electron microscopy. It seems that the expression of the mutant protein impairs the myelin of peripheral nervous system. These data might expand the genetic, clinical, and pathophysiological features of the peripheral neuropathy and a further investigation will enhance our understanding of disease in the peripheral nervous system.

## Introduction

Charcot-Marie-Tooth disease (CMT) represents a group of inherited peripheral neuropathies with a heterogeneous genetic and clinical spectrum. The typical CMT phenotype is characterized by distal weakness, sensory loss, foot deformities, and absence of reflexes [1]. CMT is broadly classified into the demyelinating type (CMT1) with slowed motor nerve conduction velocity (MNCV) of <38m/s, and the axonal defective type (CMT2) with reduced conduction velocity, >38m/s [2,3].

Although the axonal type of CMT is caused by more than 60 genes with diverse mechanisms, the demyelinating type of CMT is caused mainly by peripheral myelin proteins, the salient proteins in the myelin, or their transcription factors [4]. The most frequent genetic cause of CMT1 is alterations in *PMP22*, resulting in CMT1A or CMT1E, followed by *MPZ* mutations, which lead to CMT1B. *PMP22* and *MPZ* account for about 5% and 50% of the total peripheral myelin proteins, respectively. *PMP22* mutations account for up to 70–80% of CMT1 cases, while *MPZ* mutations occur in approximately 10% of CMT1 cases. In addition, a transcription factor for myelin proteins, *EGR2*, is the genetic cause of CMT1D [5].

Intriguingly, mutations in *Myelin basic protein* (*MBP*) and *Peripheral myelin protein 2* (*PMP2*), which account for 18% and 5% of peripheral myelin proteins, have not been reported as a genetic cause of CMT. *MBP* is also a constituent of central nervous system (CNS) myelin proteins. Although mutation in *MBP* has not been reported in humans, deletion of the MBP gene causes the ‘shiverer’ phenotype in mice, in which mice show decreased CNS myelination, tremors, and increased severity leading to early death [6].

The *PMP2* (8q21.13) was once suspected to be the causative gene because it is located in the close vicinity of the CMT4A locus (*GDAP1*) [7], but no mutation was found in the family studied [8]. To link the gene with CMT, a knockout mouse model for *PMP2* was generated; however, there was no typical phenotype of peripheral neuropathy except for a slight reduction in the nerve conduction velocity [9]. Recently, a point mutation (p.I43N) in *PMP2* was strongly suggested as a potential pathogenic mutation in a family with autosomal dominant CMT1 [10]. To demonstrate the pathogenesis of the *PMP2* mutation, the researchers showed structural abnormality caused by mutant *PMP2* expression in a zebrafish model. Several years ago, we also found a Korean CMT1 family harboring the same *PMP2* mutation and have investigated the pathogenicity of the mutation using mouse models.

In this report, we present the detailed clinical features of a *PMP2* mutation-associated autosomal dominant CMT1. In addition, the relevance of *PMP2* mutation as a genetic cause of dominant CMT1 was assessed using transgenic mouse models.

## Results

### Identification of *a PMP2* mutation

To determine the genetic cause of the FC183 family, whole exome sequencing was performed on five family members (S1 Table). The mean total sequencing yields was about 8.05 Gbp/sample, and the coverage rate of the target region (≥10X) was 91.24%. The average number of observed variants per sample was 90,653 SNPs and 6,299 indels, respectively. Of these, the number of functionally significant variants was 10,400/sample.

Exome data of the three affected members revealed ~60 functionally significant variants in CMT-related genes (S2 Table). However, none of the variants cosegregated with the affected members in the family. Most variants were polymorphic with high frequency, except for three variants with allele frequencies of less than 0.01 in Korean controls. Although three variants (p.M1I in *FAM134B*, p.A1315T in *SBF2*, and p.A952V in *CTDP1*) were not observed in in Korean controls, they were not considered as the underlying cause, because of noncosegregation with affected individuals in the FC83 family.

**Fig 1:**
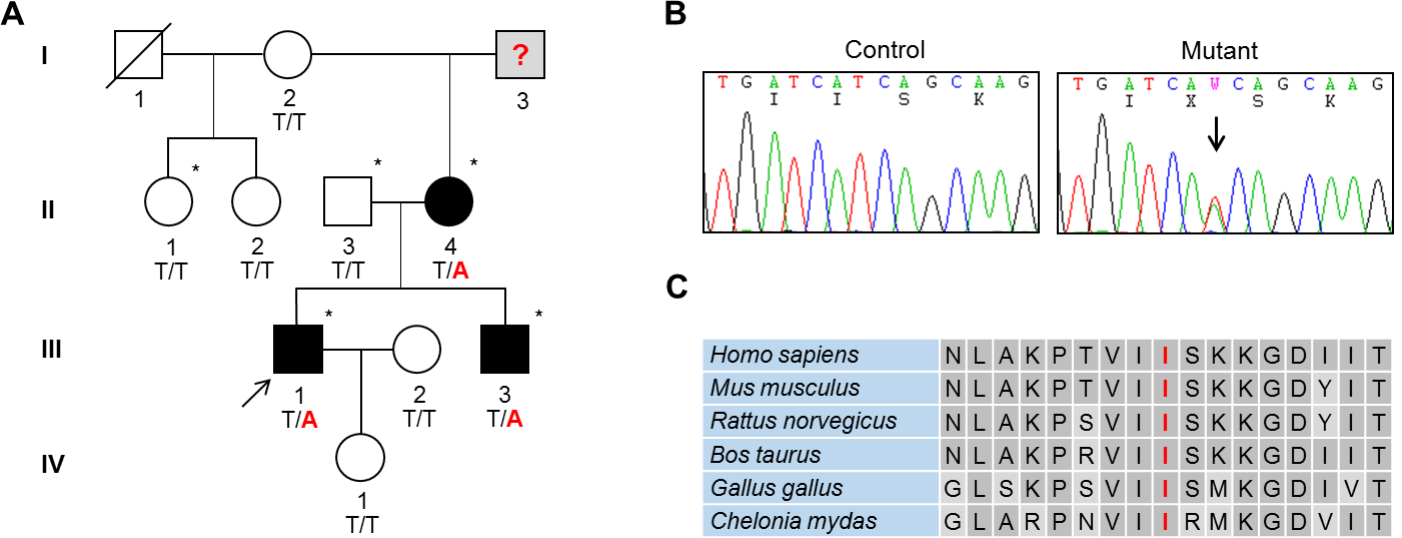
An autosomal dominant CMT 1 family with *PMP2* mutation. (A) Pedigree of the CMT 1 family (FC183). The genotype of the *PMP2* mutation is indicated at the bottom of each individual. Arrow indicates the proband. No genetic information was available for the I-3 individual. (B) Sequencing chromatogram of *PMP2* mutation. c.128T>A (p.I43N) mutation is present in *PMP2*. (C) Conservation of amino acid sequences at the mutation site in PMP2 protein. Amino acid sequences were from NP_002668.1 (*Homo sapiens*), NP_001025476.1 (*Mus musculus*), NP_001102984.1 (*Rattus norvegicus*), NP_001068707.1 (*Bos taurus*), NP_001186405.1 (*Gallus gallus*), and XP_007052997.1 (*Chelonia mydas*).

Since no variants in the CMT-related genes were considered to be an underlying cause of the CMT phenotype, we tried to identify the genetic cause from the entire exome data. A total of 5 cosegregating missense variants were isolated in *RYR2*, *NME5*, *ANK1*, *PMP2*, and *AGAP11* genes. Given that our family phenotype has a late onset, the age of the proband’s daughter (IV-1, 2 years old) would provide questionable insights into the consideration or exclusion of affection; hence she was excluded from the cosegregation analysis. All five variants were not found in the 500 Korean controls and they were not reported in global human variant databases, such as the dbSNP144, 1000G, ESP, and ExAC (Table 1). A thorough look at the variants, their gene functions, conservation, and *in silico* analysis drew attention to *PMP2* (c.T128A, p.I43N) mutation. First of all, *PMP2* encodes a myelin protein that is an important component of the peripheral nervous system. In addition, the *PMP2* mutation cosegregated within extended family members (Fig 1). The p.I43N mutation in the PMP2 protein is located in the highly conserved lipocalin/cytosolic fatty-acid binding domain, and it was predicted to be pathogenic through several *in silico* analyses using SIFT, PolyPhen2, and MUpro programs. Most of all, a recent report on the same mutation (p.I43N) also strongly suggested that the mutation is the genetic cause of this family [10].

**Table 1:** Functionally significant variants cosegregating with the affected members in the FC183 family and their *in silico* prediction.

Gene	Reference sequence^a^	Chr:position (hg19)	Variants		Affected			Unaffected				GERP^b^	*In-silico* prediction^c^		
			Nucleotide	Amino acid	II-4	III-1	III-3	I-2	II-1	II-3	III-2		SIFT	PP2	MUpro
*RYR2*	NM_001035.2	1:237870544	c.9876G>C	E3292D	G/C	G/C	G/C	G/G	G/G	G/G	G/G	0.587	0.40	0.00	0.173
*NME5*	NM_003551.2	5:137474342	c.128A>G	Q43R	A/G	A/G	A/G	A/A	A/A	A/A	A/A	5.150	0.27	0.90*	-0.186*
*ANK1*	NM_020475.2	8:41530176	c.4792C>T	H1598Y	C/T	C/T	C/T	C/C	C/C	C/C	C/C	4.180	1.00	0.01	-0.052*
*PMP2*	NM_002677.3	8:82357170	c.128T>A	I43N	T/A	T/A	T/A	T/T	T/T	T/T	T/T	5.950	0.00*	1.00*	-1.000*
*AGAP11*	NM_133447.1	10:88769232	c.1223G>A	W408X	G/A	G/A	G/A	G/G	G/G	G/G	G/G	0.149	-	-	-

^a^GenBank registration number of the reference sequence.

^b^Genomic evolutionary rate profiling (GERP) scores determined by the GERP++ program.

^c^SIFTT score <0.05, PolyPhen-2 (PP2) score ~1, and MUpro score <0 indicate a prediction of pathogenicity (*: pathogenic prediction).

### Clinical and electrophysiological manifestations

The clinical manifestations are summarized in Table 2. The age at onset ranged from 6 to 18 years among the three affected individuals. In all patients, distal leg muscle weakness, atrophy, and frequent falling were first noticed. They also exhibited bilateral hand muscle weakness that caused difficulty in writing, cooking, and fine finger control. Their hand atrophy was first noticed in the dorsal interosseous and thenar muscles, but their hypothenar muscle bulk and strength was relatively preserved.

**Table 2:** Clinical manifestations of the three patients with *PMP2* p.I43N mutation.

Patients	II-4	III-1	III-3
Sex	Female	Male	Male
Age at exam (years)	58	38	36
First symptom at onset	Distal leg muscle weakness	Frequent falling	Distal leg muscle weakness
Age at onset of leg (years)	18	6	8
Age at onset of hand (years)	41	20	28
Muscle weakness^a^			
Upper limb	+	++	+
Lower limb	+	+++	++
Muscle atrophy	Mild (U<L)	Moderate (U<L	Mild (U<L)
Sensory loss	Vibration>Pain	Vibration>Pain	Vibration>Pain
Knee/ankle jerks^b^	A/A	A/A	D/D
Plantar response	No	No	No
Hand tremor	Yes	Yes	No
Pes cavus	Yes	Yes	Yes
Scoliosis	No	No	No
FDS	3	4	3
CMTNS	9	20	13
9-HPT (sec)	22.7	31.4	26.3
Electromyography	Neurogenic MUAPs	Neurogenic MUAPs	Neurogenic MUAPs
Nerve biopsy	Demyelinating neuropathy	NE	NE

Abbreviations: A, absent; CMTNS, CMT neuropathy score; FDS, functional disability scale; NE, not examined

^a^Muscle weakness in lower limbs: + = ankle dorsiflexion 4/5 on the Medical Research Council (MRC) scale; ++ = ankle dorsiflexion <4/5 on the MRC scale; +++ = proximal weakness and wheelchair dependent. Muscle weakness in upper limbs: + = intrinsic hand weakness 4/5 on the MRC scale; ++ = intrinsic hand weakness <4/5 on the MRC scale;- = no symptoms

^b^Deep tendon reflexes: N = normal; D = diminished; A = absent

Neurological examination indicated that muscle weakness and atrophy started and predominated in the distal portions of the legs, and were noted to a lesser extent distally in the upper limbs. All of patients had pes cavus without scoliosis. Foot dorsiflexion (MRC, G0/5 to G2/5) was markedly weaker than foot plantarflexion (G4+/5 to G5/5) and finger abduction (G4/5 to G4+/5). Among the three patients, patient III-1 exhibited the most severity in muscle disability in the upper and lower limbs. All of them were able to walk independently with or without ankle-foot orthosis. Vibration sense was more severely affected than pain sense. Areflexia was noticed in the early stages of the disease, but pathologic reflexes were not found. On neurological and electrophysiological examination, her mother (I-2) showed normal findings. Based on history taking, her father (I-3) experienced no difficulty with walking.

SNAPs of the sural nerve were not detected at the early stage of the disease. Although CMAPs of the tibial motor nerves were evoked in all patients, SNAPs of sural nerves were not elicited at all (Table 3). All three patients had reduced amplitudes for the peroneal innervated foot muscles, and those responses were lesser than the responses from the thenar muscles. Needle EMG showed scattered fibrillation potentials and neurogenic motor unit action potentials.

**Table 3:** Electrophysiologic findings of the three patients with *PMP2* p.I43N mutation.

Patients	II-4	III-1	III-3	Normal values
Motor nerve conduction studies (right / left)				
Median motor nerve				
CMAPs (mV)	12.8 / 10.7	10.0 / 9.5	7.7 / 9.9	≥ 6.0
MNCVs (m/s)	21.9 / 21.6	19.4 / 16.9	15.7 / 17.3	≥ 50.5
Ulnar motor nerve				
CMAPs (mV)	14.5 / 13.2	7.9 / 9.4	13.0 / 13.0	≥ 8.0
MNCVs (m/s)	21.2 / 22.9	17.7 / 16.3	16.8 / 17.	≥ 51.1
Peroneal motor nerve				
CMAPs (mV)	NP / NP	NP / NP	NP / 0.1	≥ 1.6
MNCVs (m/s)	NP / NP	NP / NP	NP / 17.9	≥ 41.3
Tibial motor nerve				
CMAPs (mV)	3.4 / 0.9	0.2 / 0.1	1.0 / 1.6	≥ 6.0
MNCVs (m/s)	17.2 / 15.9	15.3 / 17.8	16.3 / 14.0	≥ 41.1
Sensory nerve conduction studies (right / left)				
Median sensory nerve				
SNAPs (μV)	5.4 / 4.2	NP / NP	3.1 / 6.4	≥ 8.8
SNCVs (m/s)	19.8 / 19.5	NP / NP	17.3 / 16.8	≥ 39.3
Ulnar sensory nerve				
SNAPs (μV)	5.1 / 2.4	NP / NP	6.0 / 2.3	≥ 7.9
SNCVs (m/s)	18.0 /17.2	NP / NP	12.6 / 16.8	≥ 37.5
Sural sensory nerve				
SNAPs (μV)	NP / NP	NP / NP	NP / NP	≥ 6.0
SNCVs (m/s)	NP / NP	NP / NP	NP / NP	≥ 32.1

Abbreviations: CMAP, compound muscle action potential; MNCV, motor nerve conduction velocity; NP, no potential; SNAP, sensory nerve action potential; SNCV, sensory nerve conduction velocity.

### Fatty replacement in the lower leg

**Fig 2:**
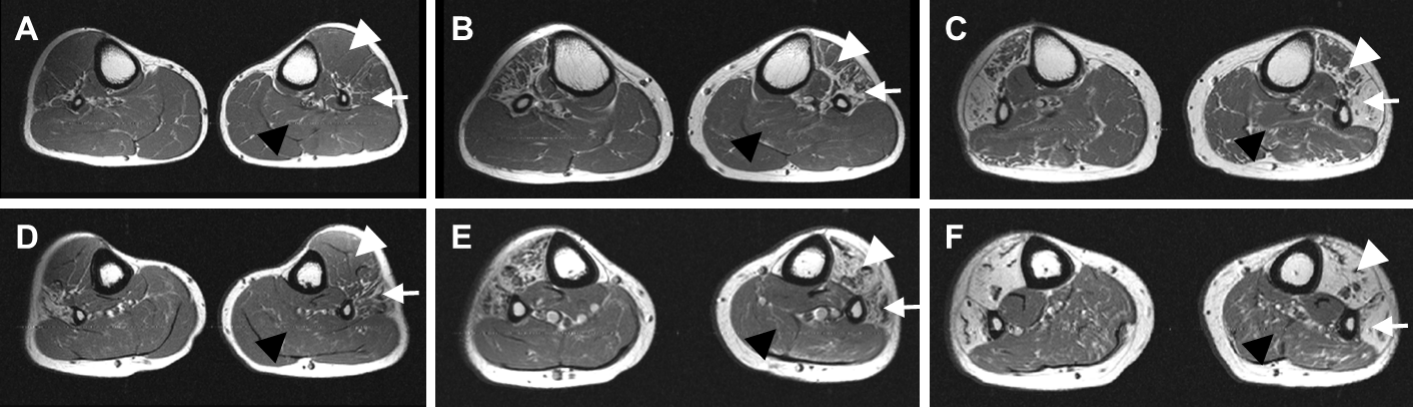
T1-weighted axial MRIs of the upper third (A-C) and lower third (D-F) calf in three patients. (A, D) III-3 patient at the age of 30 years with disease duration (DD) of 22 years. (B, E) III-1 patient at the age of 32 years with DD of 26 years. (C, F) II-4 patient at the age of 56 years with DD of 38 years. A sequential pattern of muscle involvement associated with disease duration was observed. In the early disease stage, the lateral compartment muscles (arrow) including the peroneus longus and brevis were initially involved, and they showed the most severe fatty hyperintense signal changes. A lesser degree of fatty involvement was observed in the anterior compartment muscles (white arrowhead) including the tibialis anterior, extensor hallucis longus, and extensor digitorum longus. In later stages, the posterior compartment muscles (black arrowhead) including the soleus, gastrocnemius, and tibialis posterior in the calf were relatively unaffected.

MRIs of the three patients revealed predominant signal changes in the anterior and lateral compartments of the lower leg muscles (Fig 2). T1-weighted coronal images showed more muscle atrophy and fatty replacement in the calf muscles than in the hip and thigh muscles (S1 Fig). We could observe a sequential pattern of muscle involvement associated with disease duration (Fig 2). It is noteworthy that the lateral compartment muscles including the peroneus longus and brevis were initially involved and they showed the most severe fatty hyperintense signal changes. In the early stage of the disease, diffuse fatty infiltration was observed in the lateral compartment muscles, whereas it was less severe in the anterior compartment muscles including the tibialis anterior, extensor hallucis longus, and extensor digitorum longus (Fig 2). In later stages, the posterior compartment muscles including the soleus, gastrocnemius, and tibialis posterior in the calf were still relatively unaffected (Fig 2). When we performed follow-up lower leg MRI studies at a 5-year interval, we could observe disease progression (S1 Fig). Muscle atrophy and fatty involvement in the calf were more rapid and severe than those in the thigh and hip muscles. At the hip and thigh level, the axial MR images were almost normal except for the bilateral semitendinosus and vastus lateralis muscles. At the calf level, the anterior and lateral compartment muscles showed more prominent fatty replacement than the posterior compartment muscles. In addition, brain MRI showed normal findings in the three patients (S2 Fig).

### Histopathological findings

**Fig 3:**
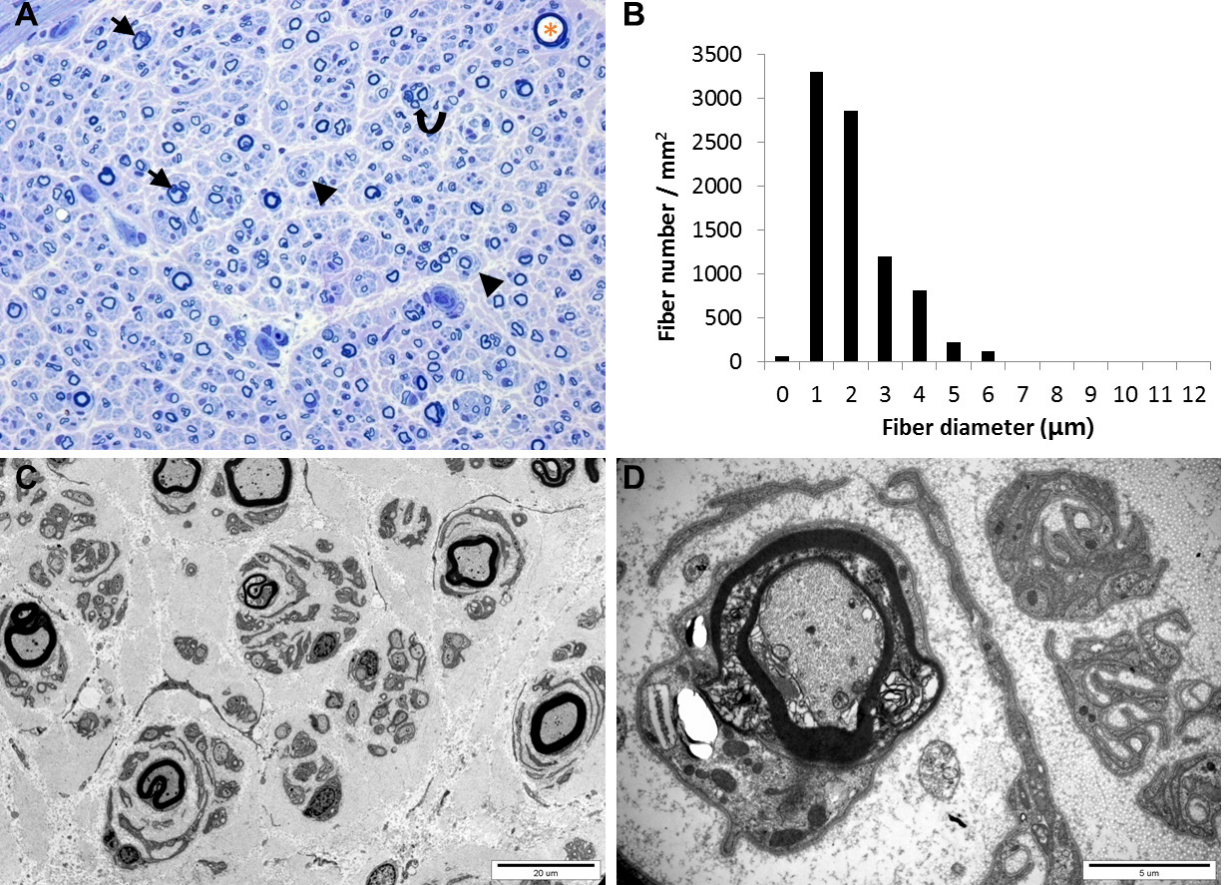
Histology of the distal sural nerve. (A) Transverse semi-thin section showed a normal range of myelinated fibers with loss of large myelinated fibers, onion bulbs (arrows), axonal degeneration with myelin abnormalities (arrow heads), and clusters of regenerative small myelinated fibers (curved arrow). (B) Histogram shows a unimodal distribution pattern. (C, D) Ultrastructural micrograph shows degenerating fibers with various abnormalities of myelin such as abnormal myelin compaction with adaxonal vacuoles, internal and external myelin folding, irregular myelin sheath, and onion bulbs or pseudo-onion bulbs surrounding the myelinated fibers. Magnifications: A, x400; C, x3000; D, x15000.

Light microscopic examination of longitudinal sections and cross sections of nerve fibers showed a slight decrease in the size of nerve fascicles as well as a decrease in the number of large myelinated fibers (MFs), and endoneurial fibrosis. Semi-thin transverse sections showed a normal range of MFs (8,601/mm^2^) with loss of large MFs, myelin abnormalities, onion bulbs, and regenerating axonal clusters (Fig 3). The histogram showed a unimodal distribution pattern of MFs. The range and average diameter of MFs were 0.86–12.61 μm and 2.59 μm, respectively. After excluding the largest MF (marked with an asterisk at the right upper corner of Fig 3A, diameter: 12.61 μm), the range and average diameter of the remaining MFs were 0.86–7.04 μm and 2.57 μm, respectively. The range and average diameter of MFs in the distal sural nerve of a normal 45-year-old female are 1.8–14.8 μm and 5.2 μm, respectively. The MF% area in this case was 5.5% (distal sural nerve of a normal 45-year-old female: 26.9%). The range and average of the g-ratio were 0.33–0.86 and 0.62±0.09, respectively (mean g-ratio in the age group of 21–50 years: 0.66). g-ratios above 0.7 comprised 18.7% of MFs and g-ratios less than 0.4 comprised 0.6% of the MFs. Electron microscopic examination showed frequent onion bulb formation, large or medium sized MFs with myelin abnormalities, regenerating axonal clusters, and thin or thick MFs (Fig 3).

### Transgenic mice exhibit peripheral neuropathy

**Fig 4:**
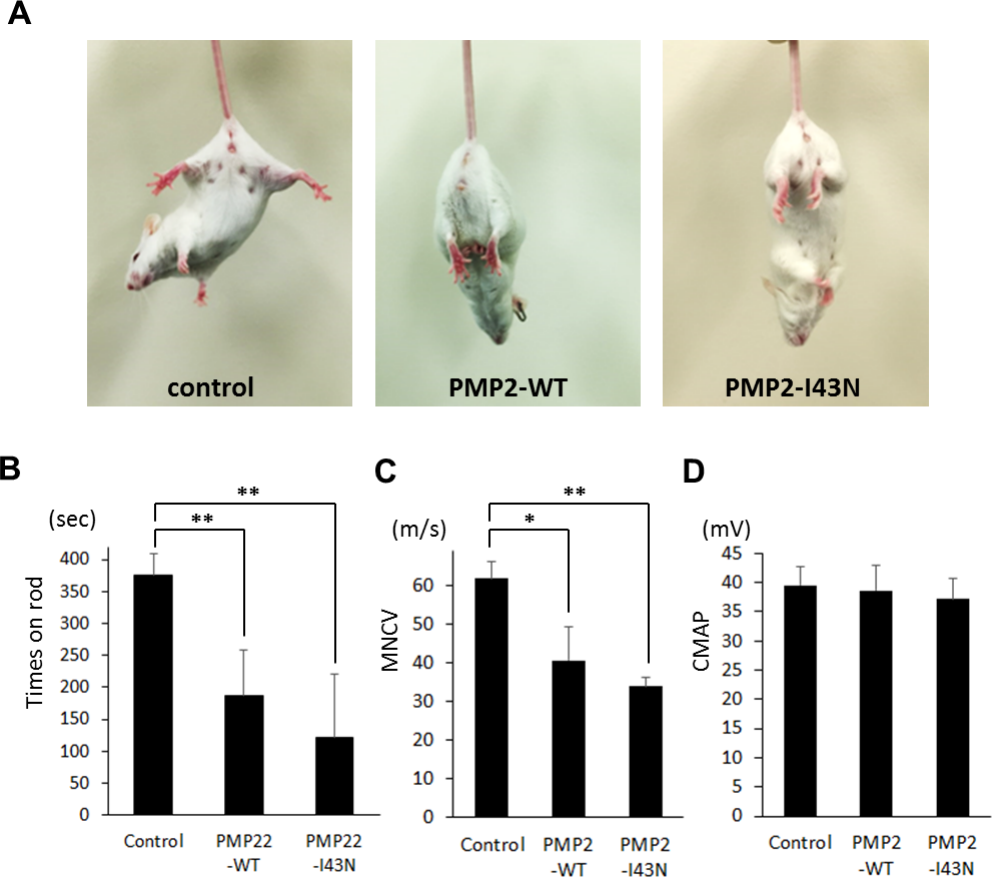
Phenotype of *PMP2* transgenic mice. (A) Tail suspension test showed hind limb folding into the abdomen in both *PMP2*-WT and *PMP2*-I43N transgenic mice. (B) Rotarod test showed significantly reduced performance in both *PMP2*-WT and *PMP2*-I43N transgenic mice at 3 months of age. (C) Reduced motor nerve conduction velocity (MNCV) in both transgenic mice. (D) Compound muscle action potential (CMAP). Ten mice from each group were used. Sweep speed, 0.5 ms per division; Amplitude, 10 mV per division. *, *p* < 0.05; and **, *p* < 0.01.

To investigate the *PMP2* mutation-associated pathogenesis, we generated transgenic mouse models for both wild type and mutant *PMP2*. Except for the peripheral neuropathy, both mice exhibited normal phenotype including normal development and breeding. To determine whether mice overexpressing human *PMP2* exhibit motor deficits, a tail suspension test was performed. Both *PMP2*-WT and *PMP2*-I43N transgenic mice showed hind limb folding into the abdomen with infrequent clasping of their hind limbs (Fig 4). Both transgenic mice also showed reduced motor performance on the rotarod test. Therefore, transgenic mice overexpressing either wild type or mutant *PMP2* definitely exhibited motor deficits compared to control mice.

To determine the nerve integrity in the hind limbs, nerve conduction was examined on both sides of the sciatic nerve at 5 months of age. Both transgenic mice showed significantly reduced MNCV compared to control mice (Fig 4). However, there was no difference in CMAP between control and *PMP2* transgenic mice. These data suggest that the phenotypes of both *PMP2* transgenic mice were similar to CMT1 rather than CMT2, which is caused by axonal defect and shows reduced CMAP.

### Demyelination and shorter internodal length in transgenic mice

**Fig 5:**
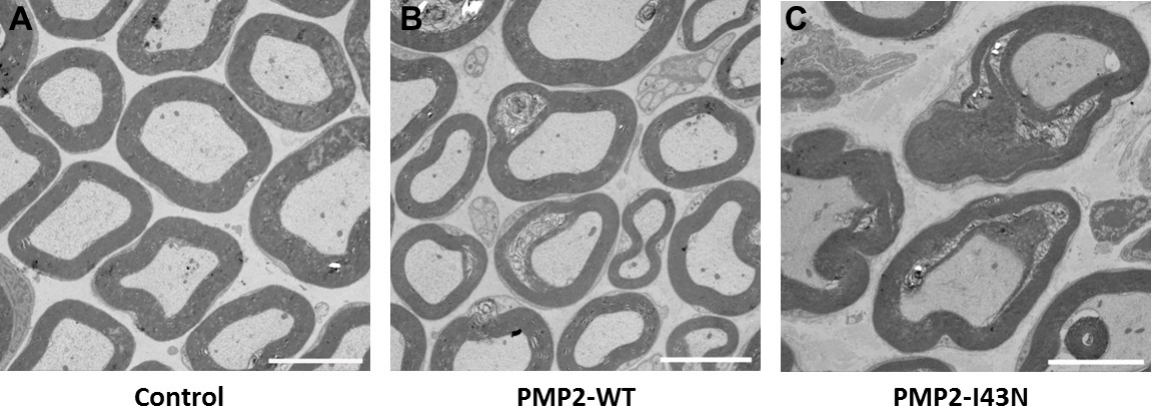
Aberration in myelination was observed in *PMP2* transgenic mice. (A) Normal control mouse. Ultrastructural micrograph showed aberrant myelination in *PMP2*-WT transgenic mouse (B) and *PMP2*-I43N transgenic mouse (C). Magnifications, x 1,500; and scale bar, 5 μm.

Semi-thin sections of the sciatic nerve displayed a reduced number of large myelinated fibers in *PMP2*-I43N mice compared to control mice, while *PMP2*-WT mice showed a pattern similar to the control mice. The average g-ratios were 0.75 ± 0.07 (control), 0.71 ± 0.09 (*PMP2*-WT), and 0.69 ± 0.09 (*PMP2*-I43N). Electron microscopic analysis revealed mixed forms of demyelinated or dysmyelinated fibers in both *PMP2*-WT and *PMP2*-I43N mice compared to control mice (Fig 5).

**Fig 6:**
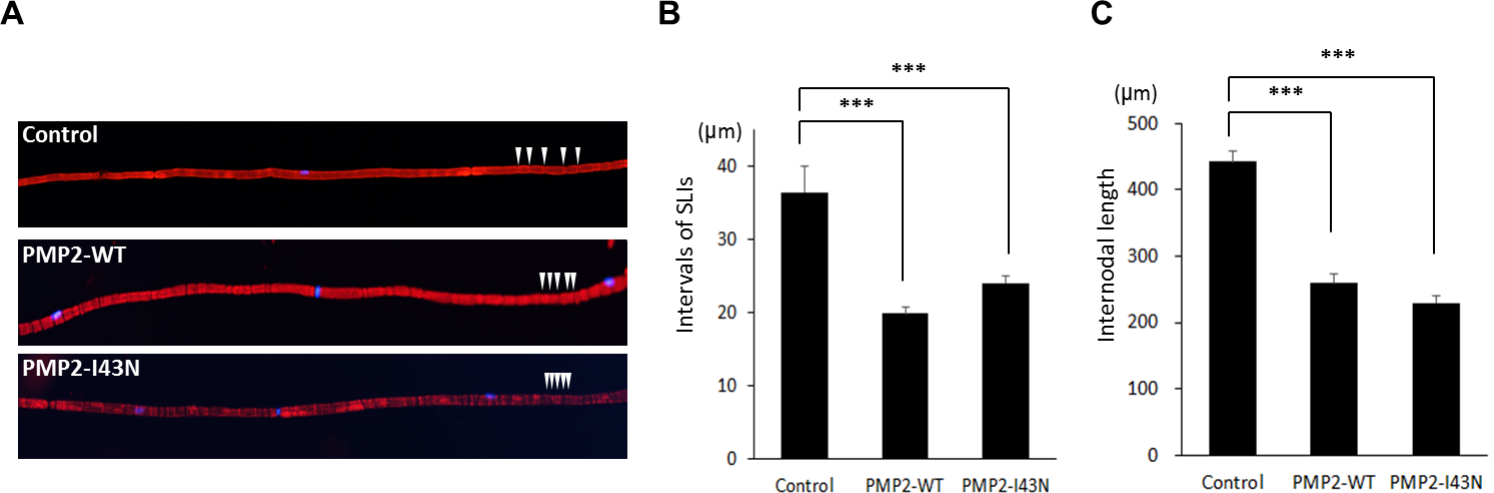
Abnormal SLI and internodal length in *PMP2* transgenic mice. (A) SLI and nuclei of Schwann cells were visualized with phalloidin (red) and DAPI (blue) staining of the teased nerve. More than 2 nuclei could be observed in the teased nerve of the transgenic mice, while only one nucleus at each field could be detected in that of control mice. SLIs were indicated as white arrow heads. (B) Intervals of SLIs were compared among the mice. Control, n = 227; *PMP2*-WT, n = 270; *PMP2*-I43N, n = 378. (C) Internodal length in each mouse model was determined by measuring the distance between nuclei (DAPI). Control, n = 164; *PMP2*-WT, n = 198; *PMP2*-I43N, n = 283. ***, *p* < 0.001.

When we analyzed the teased nerve, the *PMP2* transgenic mice revealed more frequent occurrence of Schmidt-Lanterman incisures (SLIs) (Fig 6). In addition, the mean internodal length in *PMP2*-WT and *PMP2*-I43N mice was 259.0 ± 14.6 μm and 229.3 ± 10.9 μm, which was significantly shorter than that in healthy controls (442.4 ± 15.8 μm; *P* < 0.001) (Fig 6). However, the difference in internodal length between *PMP2*-WT and *PMP2*-I43N mice was not statistically significant. Considering the shortened internodal length in the transgenic mice, the number of SLIs per myelinating Schwann cell was within a similar range of 9.6 (*PMP2*-I43N) to 13.1 (*PMP2*-WT) per cell. Collectively, these data suggest that the overexpression of either wild type or mutant *PMP2* affects Schwann cell integrity, resulting in reduced internodal length as well as demyelination, which might cause a demyelinating phenotype in transgenic mice.

## Discussion

This study strongly suggests that *PMP2* mutation is the underlying cause of an autosomal dominant CMT1 phenotype. The same mutation in *PMP2* was also suggested as a strong candidate for the cause of another CMT1 family [10]. Moreover, the pathogenicity of *PMP2* mutation had been demonstrated in a zebrafish model and the relevance of the CMT1 phenotype was also observed in our transgenic mouse models. Exome data revealed many functionally significant variants in the CMT-relevant genes from the affected individuals. Although they were not considered as the underlying cause of the CMT1 phenotype, some of them may affect clinical severity as the modifying factor(s).

The clinical features of the present family were similar to *PMP22* duplication. The age at onset among the patients was the first and second decades. The nerve conduction velocities in all three patients were below 38 m/s. Muscle weakness and atrophy started and predominated in the distal portions of the legs, and were noted to a lesser extent distally in the upper limbs. Moreover, the histopathological findings including onion bulb formation were consistent with the demyelinating type. In all patients, SNAPs of the sural nerve were not evoked at the early stage of the disease.

Additionally, there were clinical heterogeneities within the present family. The ages at onset of gait disturbance were in the first decade among the affected sons (III-1 and III-3), and in the second decade for their mother (II-4). Hand muscle weakness appeared in the third decade among the affected sons (III-1 and III-3), whereas it appeared in the fifth decade in their mother (II-4). Furthermore, the results of nerve conduction studies were mild in the mother, but were more severe in her sons.

MRI analysis revealed a distinct pattern of muscular involvement in patients with *PMP2* mutation. Marked fatty replacement was observed in the calf muscle compared to the thigh muscle, which was consistent with the hypothesis of length-dependent degeneration of motor axons. These results are useful for discerning motor neuron disease from peripheral neuropathies like CMT1 with *PMP2* mutation. In addition, we could observe a sequential pattern of muscle involvement associated with disease duration. In the early disease stage, the peronei muscles were involved, and they showed the most severe fatty replacement. The tibialis anterior muscles were also involved to a lesser degree. In the later stage, the soleus muscle in the calf was found to be relatively spared. These findings were similar to the demyelinating MR images of CMT1A patients with *PMP22* duplication, and they differed from the axonal MR images of CMT2A patients with *MFN2* mutations. Compared to CMT2A, in which severe and predominant involvement of the soleus muscles has been reported, the present patients showed relative sparing of the soleus muscles, which suggests that the etiologies and pathophysiologies of CMT2A with *MFN2* mutation are different. Therefore, MRIs of patients with *PMP2* mutation were well related to the demyelinating CMT neuropathy.

*PMP2* is a peripheral myelin protein gene that encodes a small basic P2 protein. P2 is also called M-FABP/FABP8 and is a member of a family of fatty acid binding proteins with maximal specificity of expression in the peripheral myelin [11–13]. P2 enhances myelin membrane stability and lipid dynamics [14]. P2 forms a β-barrel, which is well conserved through the species as well as throughout the FABP family subtypes with 10 anti-parallel β-strands and an N-terminal helix-loop-helix cap, where fatty acids are bound inside the barrel [15–17]. Molecular docking simulations show that the best candidate for the P2 pocket is cholesterol, one of the most abundant lipids in myelin, with a polar head that suits the electrostatic characteristics of the binding site [16]. Hydrophobic contacts surround the cavity entrance while hydrophilic contacts are located deep in the binding pocket. The mutation site (p.I43N) lies inside the pocket and substitutes a non-polar Ile with a polar Asn. Therefore, the substitution may possibly alter the dynamics of the fatty acid capacitation. *In silico* analyses also predict that *PMP2* mutation might affect protein function and stability.

The demyelinating features were also observed in *PMP2* transgenic mouse models. Both *PMP2-*WT and *PMP2-*I43N transgenic mice exhibited reduced MNCV, which is compatible with the CMT1 phenotype. We found that *PMP2* transgenic mice have shorter internodal length. Since internodal length is considered to affect the velocity of nerve impulse conduction, lower MNCV in transgenic mice might be due to the shorter internodal length [18]. Previously, shortened internodal length was reported in CMT1A patients and a *Periaxin* null mouse model [19,20]. In addition, a cell culture model using Trembler J mouse also showed reduced internodal length [21]. Therefore, shortened internodal length might be a characteristic feature of CMT1.

Since both transgenic mice either overexpressing wild type or mutant *PMP2* exhibited a similar neuropathic phenotype, we could not determine whether p.I43N mutation was pathogenic or not until the zebrafish model for *PMP2* was reported [10]. Overexpression of either wild type or mutant (p.I43N) *PMP2* in zebrafish also led to defects in axonal branching. Therefore, these data imply that *PMP2* might result in peripheral neuropathy in the same manner as the well-characterized *PMP22* gene, which affects myelination by either mutation in one allele or overdose of the wild type gene. In addition, *in vitro* assay showed that overexpression of both wild type and mutant *PMP2* in rat Schwann cell induced the markers of endoplasmic reticulum stress, which might result in cell death (S3 Fig).

Compared to the zebrafish model, our mouse models exhibited a clear CMT1 phenotype. The zebrafish model showed that aberration in *PMP2* expression exhibited motor neuron phenotypes such as axonal branching, which is somewhat different from the primary pathogenesis of CMT1. On the other hand, the zebrafish model clearly demonstrated the pathogenicity of p.I43N mutation. Overexpression of *PMP2*-I43N in zebrafish showed limited benefits compared to wild type *PMP2*. In this study, we could not directly observe the *PMP2* mutation-associated neuropathy. Phenotypic severity in the *PMP2*-I43N transgenic mouse compared to the *PMP2*-WT mouse was limited. Although *PMP2*-I43N transgenic mice exhibited worse parameters including rotarod performance, MNCV, g-ratio and internodal length than *PMP2*-WT mice, the differences were not statistically significant. For clear demonstration of *PMP2* mutation-associated neuropathy, further studies using knock-in mouse models are needed.

In this study, we first report the clinical features of patients with CMT1 caused by *PMP2* mutation as well as the characterization of transgenic mice. This report might expand the genetic and clinical features of CMT and a further mechanism study will enhance our understanding of the *PMP2* associated peripheral neuropathy.

## Materials and Methods

### Ethics statement

The study involving human patients was approved by the Institutional Review Board of Sungkyunkwan University, Samsung Medical Center (approval number: 2013-10-066). Written informed consent was obtained from all participants. All animal studies were conducted according to protocols approved by the Institutional Animal Care and Use Committees of Samsung Medical Center (approval number: 2013-080-2002).

### Patients

This study enrolled a Korean autosomal dominant CMT1 family (family ID: FC183, Fig 1) with 9 members (3 affected and 6 unaffected members) who showed no 17p12 duplication/deletion. In addition, 500 healthy controls, who had no family history of neuromuscular disorders, were enrolled after careful clinical and electrophysiological examination.

### Clinical assessment

Patients were evaluated through a detailed history including the assessment of motor impairments, sensory loss, deep tendon reflexes, and muscle atrophy. Age at onset was determined by asking the patients their ages, when symptoms such as distal muscle weakness, foot deformity, or sensory change first appeared. Muscle strengths of flexor and extensor muscles were assessed manually using the standard Medical Research Council (MRC) scale [22]. In order to determine physical disability, we used two scales, a functional disability scale (FDS) and a CMT neuropathy score (CMTNS) [23,24]. Sensory impairments were assessed in terms of the level and severity of pain, temperature, vibration and position, then pain and vibration senses were compared. The Nine-Hole Peg Test (9-HPT) was performed using both dominant and non-dominant hands; five consecutive trials for the dominant hand, followed immediately by another five consecutive trials for the non-dominant hand.

### Electrophysiological examination

Electrophysiological studies were carried out in the 3 affected individuals. Nerve conduction studies were performed by placing surface electrodes on median, ulnar, peroneal, tibial, and sural nerves. MNCVs for the median and ulnar nerves were determined by stimulating at the elbow and wrist while recording compound muscle action potentials (CMAPs) over the abductor pollicis brevis and adductor digiti quinti, respectively. In the same manner, the MNCVs of the peroneal and tibial nerves were determined by stimulating at the knee and ankle, while recording CMAPs over the extensor digitorum brevis and adductor hallucis, respectively. Sensory nerve conduction velocities (SNCVs) were obtained over a finger-wrist segment from the median and ulnar nerves by orthodromic scoring, and were also recorded for the sural nerves. Electromyography (EMG) was performed for the first dorsal interosseous, biceps brachii, tibialis anterior, medial gastrocnemius, and vastus lateralis muscles.

### Lower limb and brain MRI studies

The three patients (Fig 1: II-2, III-2, and III-4) underwent examination by lower limb MRIs of the hip, thigh, and calf muscles. MRI was performed by using a 1.5-T system (Siemens Vision, Siemens, Germany). The imaging was conducted in the axial [field of view (FOV) 24–32 cm, slice thickness 10 mm, and slice gap 0.5–1.0 mm] and coronal planes (FOV, 38–40 cm; slice thickness 4–5 mm; and slice gap 0.5–1.0 mm). The following protocol was used: T1-weighted spin-echo (SE) (TR/TE 570-650/14-20 ms), T2-weighted SE (TR/TE 2800-4000/96-99 ms), and fat-suppressed T2-weighted SE (TR/TE 3090-4900/85-99 ms). In addition, brain MRI was performed in the three patients (II-4, III-1, and III-3) by using a 1.5-T system (Siemens Vision, Siemens, Germany). Whole brains were scanned using a slice thickness of 7 mm and a 2-mm interslice gap to produce 16 axial images. The imaging protocol consisted of T2-weighted spin echo (TR/TE = 4,700/120 ms), T1-weighted spin echo (TR/TE = 550/12 ms), and fluid-attenuated inversion recovery (FLAIR) (TR/TE = 9,000/119 ms, inversion time 2,609 ms) images.

### Histopathologic studies

Patient II-4 underwent distal sural nerve biopsy at the age of 56 years. Pathological examinations included light and electron microscopic analysis. One sural nerve fragment was fixed in 10% formalin, embedded in paraffin, and stained with hematoxylin-eosin, modified Masson’s trichrome, Luxol fast blue, and Bodian stain. For the electron microscopic observation, another fragment was immediately fixed with 2% glutaraldehyde in 0.025 M cacodylate buffer at pH 7.4 and post-fixed in 1% osmium tetroxide. Epon-embedded semi-thin and ultra-thin sections were prepared for light and ultra-structural examinations.

### DNA preparation, exome sequencing and filtering

DNA was purified from peripheral blood using the QIAamp blood DNA purification kit (Qiagen, Hilden, Germany). Exome sequencing was performed on 5 members of the FC183 family (3 affecteds: II-4, III-1, III-3; and 2 unaffecteds: II-1, II-3). Exomes were captured using the SeqCap EZ ver 3.0 (Roche-NimbleGen, Madison, WI), and sequencing was performed using the HiSeq 2000 Genome analyzer (Illumina, San Diego, CA). Sequences were mapped to the human reference genome UCSC assembly hg19, and variants were called by the SAMtools program (http://samtools.sourceforge.net/). We first selected functionally significant variants (missense, nonsense, exonic indel and splicing site variants) and then filtered out polymorphic variants registered in the dbSNP (http://www.ncbi.nlm.nih.gov), 1000 Genomes project database (1000G, http://www.1000genomes.org/), Exome Sequencing Project (ESP, http://evs.gs.washington.edu/EVS/), and Exome Aggregation Consortium (ExAC, (http://exac.broadinstitute.org/). Next, we further selected variants that cosegregated with the three affected individuals within the family and were not found in the 500 healthy controls. Conservation analysis of protein sequences was conducted with MEGA ver 5.05 program [25]. Genomic evolutionary rate profiling (GERP) scores were determined with the GERP++ program (http://mendel.stanford.edu/SidowLab/downloads/gerp/index.html). *In silico* analyses were performed to predict the deleterious nature of the protein function due to amino acid substitution using SIFT (http://sift.jcvi.org/) and Polyphen-2 (http://genetics.bwh.harvard.edu/pph2/); while protein stability distortion due to amino acid substitution was predicted with the MUpro program (http://mupro.proteomics.ics.uci.edu/).

### Generation of transgenic mouse model

To obtain the human *PMP2* gene, total mRNA from HEK 293 cells was used as a template for cDNA synthesis and PCR amplification. Mutation (p.I43N) of *PMP2* was generated by site-directed mutagenesis using the QuikChange Site-Directed Mutagenesis kit (Stratagene, La Jolla, CA) and all sequences were confirmed by capillary sequencing. To establish transgenic mouse models for wild type and mutant *PMP2*, cloned vectors were injected into fertilized eggs. The eggs were implanted into surrogate female mice, then transgenic mice were generated.

### Behavior and electrophysiological examination of transgenic mice

To evaluate the motor coordination of *PMP2* transgenic mice, rotarod tests were performed on a 3 cm horizontal rotating rod at a speed of 2 m/min. To adapt to the test, mice were pre-trained for one week. For the electrophysiological test, 10 of each control and transgenic mice, aged 5 months and weighing 25–30 g, were used. The mice were anesthetized with Zoletil (50 mg/kg) intraperitoneally (Virbac, Seoul, Korea) and the fur from the distal back and the hind limbs was completely removed. The CMAP and MNCV were determined by using the Nicolet VikingQuest (Natus Medical, San Carlos, CA) as previously described [26].

### Histological examination of mice nerves

Histological analysis of mouse sciatic nerves was performed in the same manner with the patient samples. To visualize the Schmidt-Lanterman incisures (SLI) and nucleus, teased nerves were stained with rhodamine conjugated Phalloidin (Thermo Fisher Scientific, Waltham, MA) and mounted with 4′,6′-diamidine-2′-phenylindole dihydrochloride (DAPI) containing Vectashield (Vector Laboratories, Burlingame, CA) as previously reported [27]. Internodal length was determined by measuring the distance of each nuclei stained with DAPI according to a previous report [28].

### Statistical analysis

All animals were studied with a blind test. Comparison between normal and transgenic mice were made by Student’s *t*-test. P<0.05 was considered statistically significant.

## Supporting Information

S1 Table Whole exome sequencing summary for five members of the family FC183. (DOCX)

S2 Table Functionally significant variants observed in CMT causative genes in the patients of the FC183 CMT1 family. (DOCX)

S1 Fig Follow-up of lower limb MR images of III-1 patient aged 32 years (A-D) and 37 years (E-H). T1-weighted coronal (A, B, E and F), and axial (C, D, G and H) MRIs of the middle thigh (C and G), and calf (D and H) are shown. In these MRI studies at a 5-year interval, we can observe disease progression. Distal leg muscle atrophy and fatty replacement were more severe than those in proximal leg muscles. (A, C, E, and G) At the hip and thigh level, the axial MR images were almost normal in both patients except for the semitendinosus and vastus lateralis muscles. (B, D, F, and H) At the calf level, the anterior and lateral compartment muscles showed more prominent fatty replacement than the posterior compartment muscles.
(TIF)

S2 Fig Brain MRI of the III-1 patient at the age of 38 years with *PMP2* mutation showed normal findings. (TIF)

S3 Fig Cell death and endoplasmic reticulum (ER) stress induced by PMP2 proteins. (A) Cell death by overexpression of wild-type and mutant *PMP2* or *PMP22* genes were determined. Rat Schwann cell line, RT4, was transfected with indicated vectors for 72 h, then cell viability was determined using MTT (3-(4,5-dimethylthiazol-2-yl)-2,5-diphenyltetrazolium bromide) assay. The viability was displayed as % of control vector (pCMV-Myc). Data are presented as mean± SEM. *, p < 0.05. (B) Standard Western blotting exhibits induction ER stress markers, BiP and CHOP, by overexpression of wild-type and mutant *PMP2*, which were fused with Myc-epitope. RT4 cells were transfected with indicated vectors for 48 h, then expression levels of each protein were analyzed.
(TIF)
