# Supplementary material for: A Mutation in PMP2 Causes Dominant Demyelinating Charcot-Marie-Tooth Neuropathy
Source: PLoS Genet. 2016 Feb 1;12(2):e1005829. doi: 10.1371/journal.pgen.1005829 (PMC4735456; doi:10.1371/journal.pgen.1005829)
Supplement: S1 Table — (DOCX) [file pgen.1005829.s001.docx]

**S1_Table** Whole exome sequencing summary for five members of the family FC183

| Samples | Affected | | | Unaffected | |
| --- | --- | --- | --- | --- | --- |
|  | III-1 | II-4 | III-3 | II-1 | II-3 |
| Total yields (Gbp) | 5.81 | 8.42 | 10.6 | 8.83 | 6.58 |
| Mappable reads of total reads (%) | 86.5 | 85.1 | 89 | 90.1 | 91.6 |
| Coverage of the target region (≥ 1X) | 97.7 | 97.8 | 96 | 95.9 | 95.1 |
| Coverage of target region (≥ 10X) | 92.8 | 93.9 | 92.8 | 92.1 | 84.6 |
| Mean read depth of the target region | 63.4 | 95.8 | 71.8 | 52.2 | 29.2 |
| Total number of SNPs | 63,499 | 70,647 | 106,630 | 114,044 | 98,445 |
| Number of coding SNPs | 23,291 | 21,841 | 23,724 | 26,036 | 23,002 |
| Total number of indels | 3,866 | 5,923 | 8,106 | 7,680 | 5,904 |
| Number of coding indels | 295 | 340 | 345 | 338 | 323 |
