# Supplementary material for: A Mutation in PMP2 Causes Dominant Demyelinating Charcot-Marie-Tooth Neuropathy
Source: PLoS Genet. 2016 Feb 1;12(2):e1005829. doi: 10.1371/journal.pgen.1005829 (PMC4735456; doi:10.1371/journal.pgen.1005829)
Supplement: S2 Table — (DOCX) [file pgen.1005829.s002.docx]

**S2_Table** Functionally significant variants observed in CMT causative genes in the patients of the FC183 CMT1 family

| Genes | Reference sequences^a^ | Variants | | dbSNP144 | Variant allele frequencies | | | |  | *In silico* analysis^f^ | | Affected | | | Unaffected | |
| --- | --- | --- | --- | --- | --- | --- | --- | --- | --- | --- | --- | --- | --- | --- | --- | --- |
|  |  | Nucleotide | Amino acid |  | Korea^b^ | 1000G^c^ | ExAC^c^ | ESP^c^ |  | PP2 | MU | III-1 | II-4 | III-3 | II-1 | II-3 |
| *PLEKHG5* | NM_020631.4 | c.260T>C | p.I87T | rs117505788 | 0.065 | 0.008 | 0.0036 | 0.0002 |  | 0.77 | -0.76 | +/− | +/− | −/− | −/− | −/− |
| *HADHB* | NM_000183.2 | c.427C>A | p.Q143K | rs200718690 | 0.079 | N | 0.0469 | N |  | 0.76 | 0.966 | −/− | +/− | −/− | +/− | −/− |
|  |  | c.3_4insACT | p.M1delinsMT | rs147970487 | 0.164 | 0.710 | 0.8315 | 0.0868 |  | − | − | +/− | −/− | −/− | −/− | +/− |
| *SCN9A* | NM_002977.3 | c.3448T>C | p.R1150W | rs6746030 | 0.509 | 0.892 | 0.8693 | 0.8774 |  | 0.94 | -0.929 | +/+ | +/+ | +/+ | +/+ | +/+ |
| *OPA1* | NM_130834.2 | c.473G>A | p.S158N | rs7624750 | 0.335 | 0.532 | 0.4683 | 0.4970 |  | 0.00 | -0.704 | +/+ | +/− | +/− | +/+ | +/− |
| *FAM134B* | NM_019000.4 | c.3G>A | p.M1I | rs754693880 | N | N | 0.0001 | N |  | 0.91 | -0.591 | +/− | −/− | +/− | −/− | +/− |
| *DST* | NM_015548.4 | c.8439G>A | p.M2813I | rs4715630 | 0.498 | 0.792 | 0.7601 | 0.7754 |  | 0.00 | 0.600 | +/+ | +/+ | +/+ | +/− | +/+ |
|  |  | c.8176A>G | p.T2726A | rs4715631 | 0.468 | 0.668 | 0.7040 | 0.6829 |  | 0.01 | -0.936 | +/− | +/− | +/− | +/+ | +/+ |
|  |  | c.6872G>A | p.R2291H | rs11756977 | 0.265 | 0.336 | 0.2982 | 0.7662 |  | 0.00 | -0.862 | +/− | +/− | −/− | +/− | −/− |
|  |  | c.6301T>C | p.S2101P | rs148547958 | 0.023 | 0.005 | 0.0017 | 0.0001 |  | 0.93 | 0.576 | −/− | +/− | +/− | −/− | −/− |
|  |  | c.3923A>G | p.Q1308R | rs4712138 | 0.225 | 0.328 | 0.3562 | 0.6123 |  | 0.01 | 0.753 | +/− | +/+ | +/− | +/− | −/− |
|  |  | c.6031G>C | p.V2011L | rs6459166 | 0.237 | 0.402 | 0.3887 | 0.6189 |  | 0.00 | -0.856 | +/− | +/+ | +/− | +/− | −/− |
|  |  | c.98T>C | p.L33S | rs1024196 | 0.023 | 0.132 | 0.0611 | 0.1602 |  | 0.00 | -0.991 | −/− | +/− | +/− | −/− | −/− |
| *FIG4* | NM_014845.5 | c.1090A>T | p.M364L | rs2295837 | 0.236 | 0.100 | 0.0743 | 0.0309 |  | 0.03 | -0.964 | −/− | +/− | −/− | +/− | −/− |
| *GARS* | NM_002047 | c.124C>G | p.P42A | rs1049402 | 0.390 | 0.654 | 0.7030 | 0.7399 |  | 0.00 | -0.805 | +/− | +/− | +/− | +/− | −/− |
| *ARHGEF10* | NM_014629.2 | c.1110G>C | p.L370F | rs9657362 | 0.219 | 0.168 | 0.1594 | 0.1081 |  | 0.71 | -0.835 | +/− | −/− | +/− | −/− | +/− |
| *IKBKAP* | NM_003640.3 | c.3473C>T | p.P1158L | rs1538660 | 0.263 | 0.245 | 0.1988 | 0.2119 |  | 0.01 | -0.709 | −/− | +/− | −/− | +/− | −/− |
|  |  | c.3214T>A | p.C1072S | rs3204145 | 0.263 | 0.246 | 0.1984 | 0.2125 |  | 0.00 | -0.893 | −/− | +/− | −/− | +/− | −/− |
|  |  | c.2446A>C | p.I816L | rs2230793 | 0.288 | 0.298 | 0.2271 | 0.2706 |  | 0.00 | -0.999 | −/− | +/− | −/− | −/− | −/− |
|  |  | c.2294G>A | p.G765E | rs2230792 | 0.274 | 0.289 | 0.2306 | 0.2605 |  | 0.00 | -0.506 | −/− | +/− | −/− | −/− | −/− |
| *LRSAM1* | NM_138361.5 | c.952A>G | p.N318D | rs1539567 | 0.466 | 0.727 | 0.7776 | 0.7418 |  | 0.00 | 0.569 | +/+ | +/− | +/+ | +/+ | +/+ |
| *SETX* | NM_015046.5 | c.7834A>G | p.S2612G | rs3739927 | 0.330 | 0.164 | 0.0852 | 0.0730 |  | 0.00 | -0.785 | +/− | +/− | −/− | −/− | −/− |
|  |  | c.7759A>G | p.I2587V | rs1056899 | 0.482 | 0.539 | 0.3926 | 0.4442 |  | 0.00 | -0.629 | +/+ | +/− | +/− | +/− | +/+ |
|  |  | c.5563A>G | p.T1855A | rs2296871 | 0.477 | 0.443 | 0.2758 | 0.3068 |  | 0.00 | -0.889 | +/+ | +/− | +/− | +/− | +/+ |
|  |  | c.4156A>G | p.I1386V | rs543573 | 0.242 | 0.556 | 0.7252 | 0.6938 |  | 0.00 | -0.935 | −/− | +/− | +/− | +/− | −/− |
|  |  | c.3754G>A | p.G1252R | rs1183768 | 0.231 | 0.556 | 0.7251 | 0.6931 |  | 0.48 | 0.905 | −/− | +/− | +/− | +/− | −/− |
|  |  | c.3576T>G | p.D1192E | rs1185193 | 0.256 | 0.641 | 0.7635 | 0.7929 |  | 0.01 | -0.745 | −/− | +/− | +/− | +/− | −/− |
|  |  | c.3455T>G | p.F1152C | rs3739922 | 0.214 | 0.090 | 0.0656 | 0.0384 |  | 0.01 | -0.838 | +/− | −/− | +/− | +/− | +/+ |
|  |  | c.1979C>G | p.A660G | rs882709 | 0.317 | 0.214 | 0.1211 | 0.1138 |  | 0.42 | -0.999 | +/− | −/− | +/− | +/− | +/− |
| *DHTKD1* | NM_018706.6 | c.814T>G | p.Y272D | rs3740015 | 0.380 | 0.484 | 0.5596 | 0.4801 |  | 0.00 | -0.918 | +/+ | +/+ | +/− | +/− | +/− |
|  | NM_018706.6 | c.1821C>G | p.I607M | rs2062988 | 0.469 | 0.727 | 0.7788 | 0.7420 |  | 0.00 | -0.852 | +/+ | +/+ | +/+ | +/− | +/+ |
| *HK1* | NM_033500.2 | c.20A>G | p.H7R | rs906220 | 0.509 | 0.905 | 0.9102 | 0.8972 |  | 0.01 | -0.509 | +/+ | +/+ | +/+ | +/+ | +/+ |
| *SBF2* | NM_030962.3 | c.3943G>A | p.A1315T | N | N | N | N | N |  | 0.09 | -0.956 | +/− | −/− | +/− | −/− | +/− |
| *IGHMBP2* | NM_002180.2 | c.602T>C | p.L201S | rs560096 | 0.429 | 0.687 | 0.7719 | 0.7786 |  | 0.00 | -0.991 | +/+ | +/+ | +/+ | +/− | +/+ |
|  |  | c.2011A>G | p.T671A | rs622082 | 0.170 | 0.246 | 0.2988 | 0.2468 |  | 0.00 | -0.523 | −/− | +/− | +/− | +/− | −/− |
|  |  | c.2636C>A | p.T879K | rs17612126 | 0.311 | 0.197 | 0.2498 | 0.2101 |  | 0.22 | 0.567 | +/+ | +/− | +/− | −/− | +/+ |
| *MTMR2* | NM_016156 | c.1805C>G | p.A602G | rs76784113 | 0.036 | 0.005 | 0.0022 | N |  | 0.14 | -0.844 | −/− | +/− | +/− | −/− | −/− |
| *WNK1* | NM_213655.4 | c.3922A>C | p.T1308P | rs956868 | 0.500 | 0.841 | 0.8314 | 0.8526 |  | 0.00 | -0.525 | +/+ | +/+ | +/+ | +/− | +/− |
|  |  | c.5273G>C | p.C1758S | rs7955371 | 0.509 | 0.987 | 0.9963 | 0.9861 |  | 0.00 | -0.693 | +/+ | +/+ | +/+ | +/+ | +/+ |
|  |  | c.6180G>T | p.M2060I | rs12828016 | 0.251 | 0.418 | 0.3766 | 0.4314 |  | 0.00 | -0.926 | +/− | −/− | +/− | −/− | +/+ |
|  |  | c.6589C>T | p.R2197C | rs117016551 | 0.019 | 0.002 | 0.0008 | N |  | 1.00 | -0.853 | −/− | +/− | −/− | −/− | −/− |
| *DYNC1H1* | NM_001376.4 | c.7463G>A | p.R2488H | rs149581331 | 0.007 | 0.002 | 0.0007 | N |  | 0.01 | -0.937 | +/− | +/− | +/− | +/− | −/− |
|  |  | c.7979T>G | p.V2660G | N | 0.093 | N | N | N |  | 1.00 | -0.993 | +/− | −/− | −/− | −/− | +/− |
| *SEPT9* | NM_001113495.1 | c.1390A>G | p.M464V | rs2627223 | 0.507 | 0.914 | 0.9371 | 0.8923 |  | 0.00 | -0.936 | +/+ | +/+ | +/+ | +/+ | +/+ |
| *CTDP1* | NM_004715.4 | c.2855C>T | p.A952V | rs757157825 | N | N | 0.0001 | N |  | 1.00 | -0.612 | +/− | −/− | −/− | −/− | +/− |
| *DNMT1* | NM_001379.2 | c.931A>G | p.I311V | rs2228612 | 0.305 | 0.177 | 0.132 | 0.0863 |  | 0.00 | -0.749 | +/− | +/− | +/− | +/− | +/− |
|  | NM_001379.2 | c.290A>G | p.H97R | rs16999593 | 0.142 | 0.045 | 0.0217 | 0.0015 |  | 0.42 | 0.720 | +/− | −/− | +/− | +/− | +/− |
| *DNM2* | NM_001190716.1 | c.1958T>G | p.V653G | rs201780856 | 0.296 | N | 0.3366 | N |  | 0.99 | -0.945 | +/− | +/− | −/− | −/− | +/− |
| *PRX* | NM_181882.2 | c.3394G>A | p.G1132R | rs268674 | 0.509 | 0.956 | 0.9469 | 0.9538 |  | 0.00 | -0.622 | +/+ | +/+ | +/+ | +/− | +/+ |
|  |  | c.2763A>G | p.I921M | rs268673 | 0.099 | 0.297 | 0.3504 | 0.3663 |  | 0.00 | -0.752 | −/− | +/− | −/− | −/− | −/− |
|  |  | c.2611G>C | p.V871L | N | 0.064 | N | N | N |  | 0.40 | 0.525 | +/− | +/− | +/− | +/− | −/− |
| *ATP7A* | NM_000052.5 | c.4048G>A | p.E1350K | rs4826245 | 1.000 | 1.000 | 1 | N |  | 0.89 | -0.989 | + | +/+ | + | +/+ | + |

+: mutation, -: no mutation, N: no report.

^a^GenBank registration number of the reference sequence.

^b^Generated by our laboratory using 302 whole exome sequencing data.

^c^Variant allele frequencies in the 1000 Genomes Project (1000G), the Exome Aggregation Consortium (ExAC), and the Exome Sequencing Project (ESP).

^d^*In silico* scores of PolyPhen-2 (PP2) ~1 and MUpro (MU) <0 predict the affection of protein function or stability.
