# Supplementary figures and images for: A Mutation in PMP2 Causes Dominant Demyelinating Charcot-Marie-Tooth Neuropathy
Source: PLoS Genet. 2016 Feb 1;12(2):e1005829. doi: 10.1371/journal.pgen.1005829 (PMC4735456; doi:10.1371/journal.pgen.1005829)

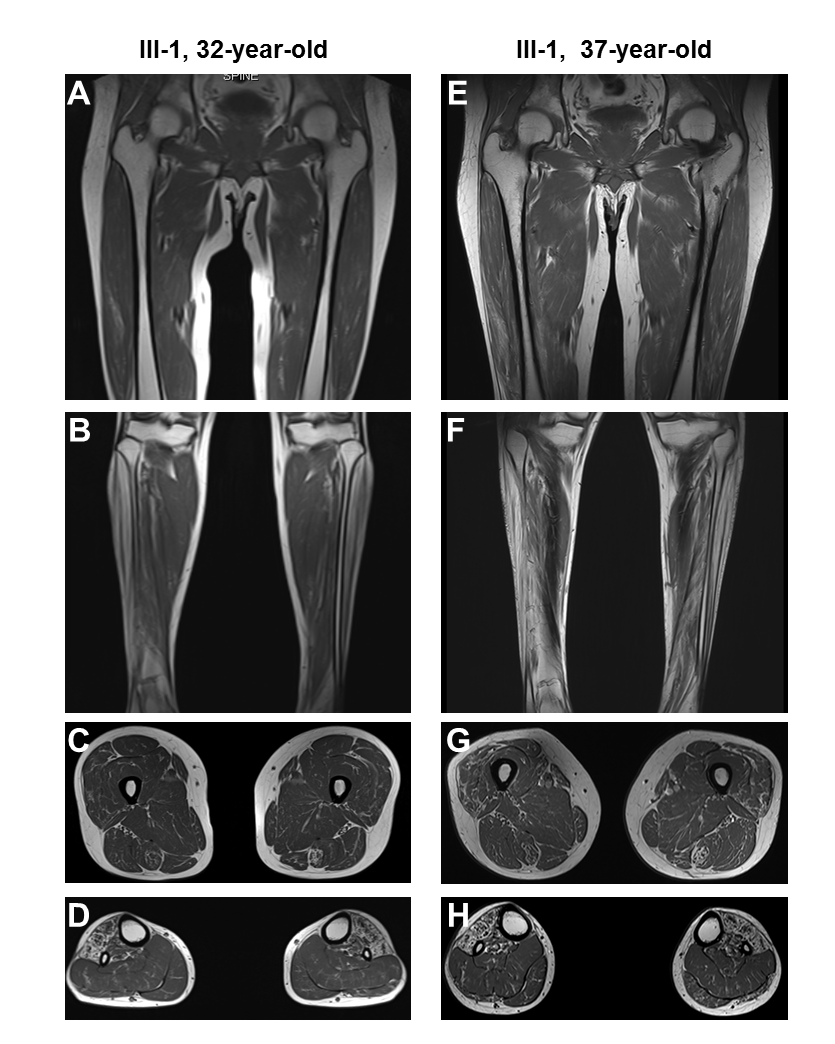

Supplement: S1 Fig — T1-weighted coronal (A, B, E and F), and axial (C, D, G and H) MRIs of the middle thigh (C and G), and calf (D and H) are shown. In these MRI studies at a 5-year interval, we can observe disease progression. Distal leg muscle atrophy and fatty replacement were more severe than those in proximal leg muscles. (A, C, E, and G) At the hip and thigh level, the axial MR images were almost normal in both patients except for the semitendinosus and vastus lateralis muscles. (B, D, F, and H) At the calf level, the anterior and lateral compartment muscles showed more prominent fatty replacement than the posterior compartment muscles. (TIF) [file pgen.1005829.s003.tif]

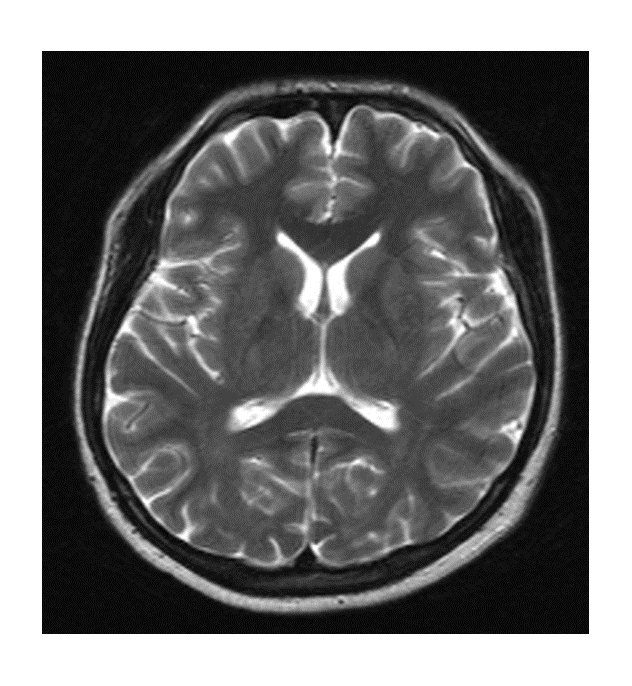

Supplement: S2 Fig — (TIF) [file pgen.1005829.s004.tif]

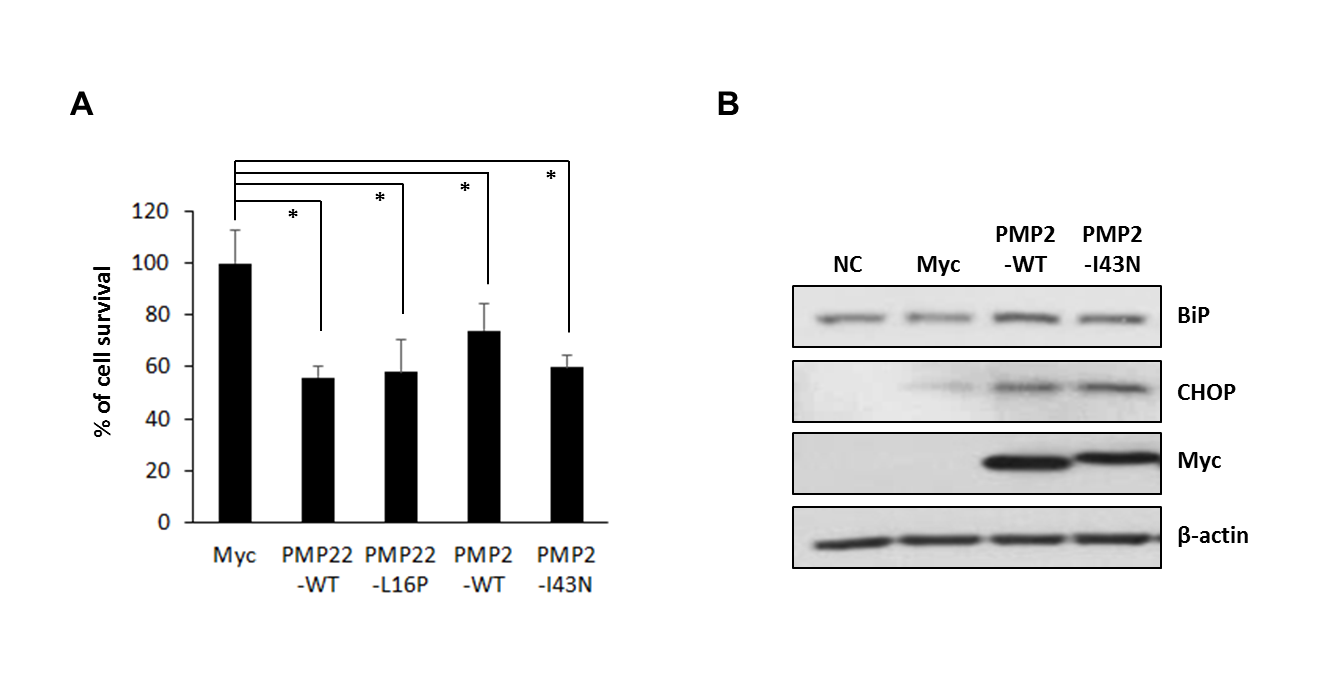

Supplement: S3 Fig — (A) Cell death by overexpression of wild-type and mutant PMP2 or PMP22 genes were determined. Rat Schwann cell line, RT4, was transfected with indicated vectors for 72 h, then cell viability was determined using MTT (3-(4,5-dimethylthiazol-2-yl)-2,5-diphenyltetrazolium bromide) assay. The viability was displayed as % of control vector (pCMV-Myc). Data are presented as mean± SEM. *, p < 0.05. (B) Standard Western blotting exhibits induction ER stress markers, BiP and CHOP, by overexpression of wild-type and mutant PMP2, which were fused with Myc-epitope. RT4 cells were transfected with indicated vectors for 48 h, then expression levels of each protein were analyzed. (TIF) [file pgen.1005829.s005.tif]
